# New Insights into Metabolic Properties of Marine Bacteria Encoding Proteorhodopsins

**DOI:** 10.1371/journal.pbio.0030273

**Published:** 2005-07-19

**Authors:** Gazalah Sabehi, Alexander Loy, Kwang-Hwan Jung, Ranga Partha, John L Spudich, Tal Isaacson, Joseph Hirschberg, Michael Wagner, Oded Béjà

**Affiliations:** **1**Department of Biology, Technion-Israel Institute of Technology, Haifa, Israel,; **2**Department of Microbial Ecology, University of Vienna, Austria,; **3**Center for Membrane Biology, Department of Biochemistry and Molecular Biology, The University of Texas Medical School, Houston, Texas, United States of America,; **4**Department of Genetics, The Hebrew University of Jerusalem, Israel; Institute for Genomic ResearchUnited States of America

## Abstract

Proteorhodopsin phototrophy was recently discovered in oceanic surface waters. In an effort to characterize uncultured proteorhodopsin-exploiting bacteria, large-insert bacterial artificial chromosome (BAC) libraries from the Mediterranean Sea and Red Sea were analyzed. Fifty-five BACs carried diverse proteorhodopsin genes, and we confirmed the function of five. We calculate that proteorhodopsin-exploiting bacteria account for 13% of microorganisms in the photic zone. We further show that some proteorhodopsin-containing bacteria possess a retinal biosynthetic pathway and a reverse sulfite reductase operon, employed by prokaryotes oxidizing sulfur compounds. Thus, these novel phototrophs are an unexpectedly large and metabolically diverse component of the marine microbial surface water.

## Abstract 2

Metagenomic analyses of the Mediterranean Sea and Red Sea estimate that proteorhodopsin genes are exploited by a surprisingly high percentage of bacteria. Experimental studies reveal metabolically diverse roles for these phototrophs.

## Introduction

Proteorhodopsin (PR) proteins are bacterial retinal-binding membrane pigments that function as light-driven proton pumps in the marine ecosystem [1,2]. A gene encoding such a pigment was originally discovered on a large genome fragment [1] derived from an uncultured marine gammaproteobacterium of the SAR86 group [3,4]. Subsequently, many diverse PRs have been detected in marine plankton, via PCR-based gene surveys [5,6], environmental bacterial artificial chromosome (BAC) and fosmid libraries screening [7,8], or environmental shotgun libraries [9]. Recently, through comparative analyses of SAR86 rRNA-bearing genomic fragments, it was shown that diverse SAR86 members contain PR pigments belonging to different groups [7]. Furthermore, in another environmental genomics study, it was proposed that a Pacific PR is encoded by a planktonic alphaproteobacterium [8]. Although retrieval and comparative analyses of large genome fragments carrying PR genes is the most promising approach to phylogenetically assign and better understand uncultured PR-carrying organisms, the data accumulated to this day come from only five different PR genes contained within large insert BAC or fosmid clones: the original Pacific 31A08 clone [1], Antarctic ANT32C12 fosmid clone [8], Pacific *Alphaproteobacteria*-related clone HOT2C01 [8], Pacific clone HOT4E07, and eBAC20E09 clone from the Red Sea [7].

## Results/Discussion

**Figure 1:**
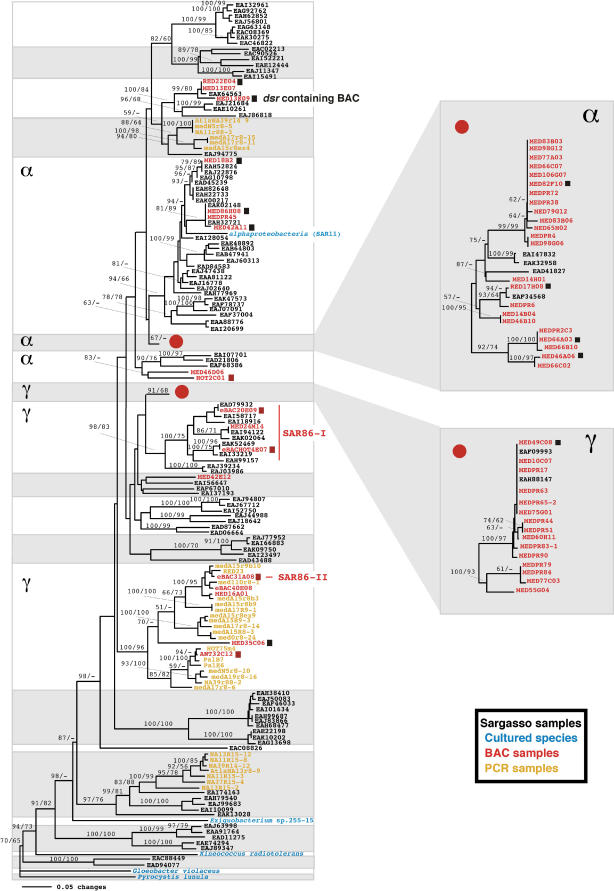
Phylogenetic Tree of PR Proteins from the Mediterranean and Red Seas along with PR Homologs in GenBank The tree was divided into what we propose are distinct subfamilies of sequences, based on bootstrap values significance. The tree was constructed as follows: (i) All homologs of PR proteins were identified in GenBank including predicted proteins from the Sargasso Sea assemblies using BLASTp [36] searches with representatives of previously identified PR-like protein families as query sequences. (ii) All sequences greater than 300 nucleotides in length were aligned to each other using CLUSTALx [37], and a neighbor-joining phylogenetic tree was inferred using the neighbor programs of PAUP* [38]. Bootstrap resampling (1,000 pseudoreplications) of neighbor-joining and maximum parsimony trees were performed in all analyses to provide confidence estimation for the inferred topologies. Bootstraps values greater than 50% are indicated above the branches (neighbor-joining/maximum parsimony). The scale bar represents the number of substitutions per site. The sequences are colored according to the type of sample in which they were found: blue, cultured species; orange, sequences from uncultured organisms obtained using PCR-based methods; and red, BAC-derived sequences from uncultured species in the Mediterranean Sea and Red Sea (this study) or from previously reported Pacific, Antarctic, and Red Sea [1,7,8] BAC/fosmids. Black squares mark sequenced BACs in this study; red squares label BACs sequenced in previous reports. α, *Alphaproteobacteria*; γ, *Gammaproteobacteria*. Red circles mark the two abundant PR groups discussed in the manuscript.

To better understand the extent of naturally occurring PR variability and physiological traits associated with PR-carrying organisms, we surveyed large insert BAC libraries (with inserts up to 170 Kb) from the photic zone of the Mediterranean Sea and Red Sea using Southern hybridization and newly designed general degenerated PR primers. The primers were designed based on alignments of PR sequences from the North Atlantic Ocean, the Mediterranean and Red Seas [5,6], the Pacific Ocean [7,8], and from the Sargasso Sea environmental shotgun project [9]. These primers amplified diverse PR sequences (red in Figure 1), which were not restricted to the three PR families we previously amplified using non-degenerative primers (orange in Figure 1). The diversity of PRs observed in the BAC library was comparable to recent findings from randomly sequenced small-insert shotgun libraries from the Sargasso Sea [9]. Fifty-five different BAC clones were found to contain PRs in the Mediterranean library, representing 0.52% of the total clones. Assuming (i) that an average marine bacterium had a genome size of 2.0 Mb, (ii) that the cloned DNA was recovered from exclusively prokaryotes, and (iii) that each PR-carrying microorganism carried only one PR gene copy on its genome, this PR abundance suggests that 13% of the bacteria in the photic zone of the Mediterranean Sea possess a PR gene (10,560 BAC clones × 80-Kb average insert size = 844.8 Mb; 844.8 Mb / 2.0 Mb = 422.2 genomes represented in the library; 55 PR genes in 422.2 genomes represent 13%). This estimation does not consider possible biases of environmental BAC libraries prepared in *Escherichia coli* against foreign DNA [10]. Interestingly, 50% of these PR-containing BAC clones fall into two distinct groups (red circles in Figure 1), which might represent the most abundant PR-containing bacteria in Mediterranean surface waters.

**Figure 2:**
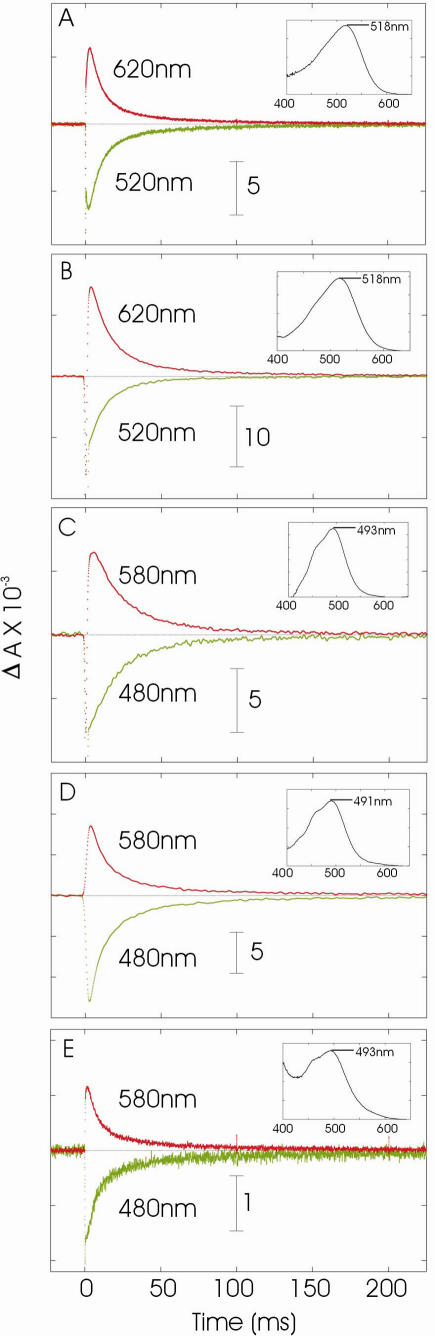
Laser-Flash-Induced Absorbance Changes in Suspensions of *E. coli* Membranes Containing PR Proteins (A–E) PR proteins are from BACs MED46A06, MED66A03, MED18B02, MED49C08, and MED13K09, respectively. A 532-nm pulse (6-ns duration, 40 mJ) was delivered at time 0, and absorption changes were monitored at wavelengths near the absorption maximum of the main absorption band in the visible range of the unphotolyzed pigment (520 nm for A and B, 480 nm for C–E) and the final photointermediate (the O intermediate) which is the longest-lived species in each of the photochemical reaction cycles (620 nm for A and B, 580 nm for C–E). 150–2,000 transients were collected at one to two flashes/sec and averaged for each trace as previously described [39]. The bar in each panel indicates the scale of the absorption change (× 10^−3^). Panel E exhibits greater noise because of the lower amplitudes of absorption changes due to lower expression level of the pigment. Insets: *E. coli* membranes containing PR apoproteins in 50 mM Tris-HCl (pH 9.0) were reconstituted with an ethanolic solution of 2 μM all-*trans* retinal. The retinal-reconstitution of PR pigments were recorded in a Cary 4000 spectrophotometer at room temperature. The spectra were taken 40 min after retinal addition, which produced between 0.035 to 0.078 absorption units at the absorption maxima indicated.

BAC clones representing each PR family (black squares in Figure 1) were partially or completely sequenced and annotated (11 clones in total). Two and seven out of these 11 BAC clones are suggested to be coming from prokaryotes related to the *Gamma*- and *Alphaproteobacteria*, respectively, based on top BLAST hits criteria (see Tables S1–S6) and previously published information [8]. Based on homology searches, we were able to assign BAC clone MED49C08 from one of the gammaproteobacterial groups to the SAR86 clade; thus, 14 other BAC clones with almost identical PR genes (Figure 1) were also considered as members of this group (assuming no lateral gene transfer in the case of PR). Three of the retrieved BAC clones (MED86H08, MEDPR45, and MED42A11) are predicted to be from the SAR11 group because they carry PR genes with high sequence homology to a PR recently identified by proteome analysis of a cultured alphaproteobacterium (SAR11) [11], and data from other genes on the BACs support alphaproteobacterial affiliations. The high abundance of genome fragments from SAR86 and *Alphaproteobacteria* found here is consistent with previous reports, which determined members of the SAR86 clade to account for up to 8% of the active bacteria in the photic zone of a coastal North Sea sample [3] while SAR11 members were found to represent as much as 50% of the total marine surface water microbial community [12]. Based on 16S rRNA surveys, both the SAR86 and SAR11 clades harbor very diverse populations [13]. This “microdiversity” is also reflected on the PR level (Figure 1). All PR representatives (*Alphaproteobacteria* MED18B02, MED46A06, MED66A3; *Gammaproteobacteria* MED49C08; and unassigned group MED13K09 and MED82F10) checked using the *E. coli* heterologous expression system showed light-driven proton pumping activity as well as fast photocycles typical of retinylidene transporters [14] (Figure 2). The photochemical reaction cycles observed are among the most rapid seen for proton-pumping rhodopsins. Of interest is that the pigments exhibiting blue absorption spectra (MED18B02, MED49C08, and MED13K09) have fast photocycles indicative of efficient proton pumps operating in a high solar radiation environment as found in surface water (12-m depth) from which the BAC library was prepared. In contrast, the only previously characterized blue absorbing PR, HOT75 [15], has an order-of-magnitude slower photocycle. This was previously attributed to its retrieval from 75-m depth, where solar flux intensities are greatly reduced [15]. Taken together, these data imply that the widespread marine SAR86 and SAR11 groups, as well as other bacterial groups, are using light-driven PR-based phototrophy as a way to harvest additional energy in oligotrophic marine environments.

**Figure 3:**
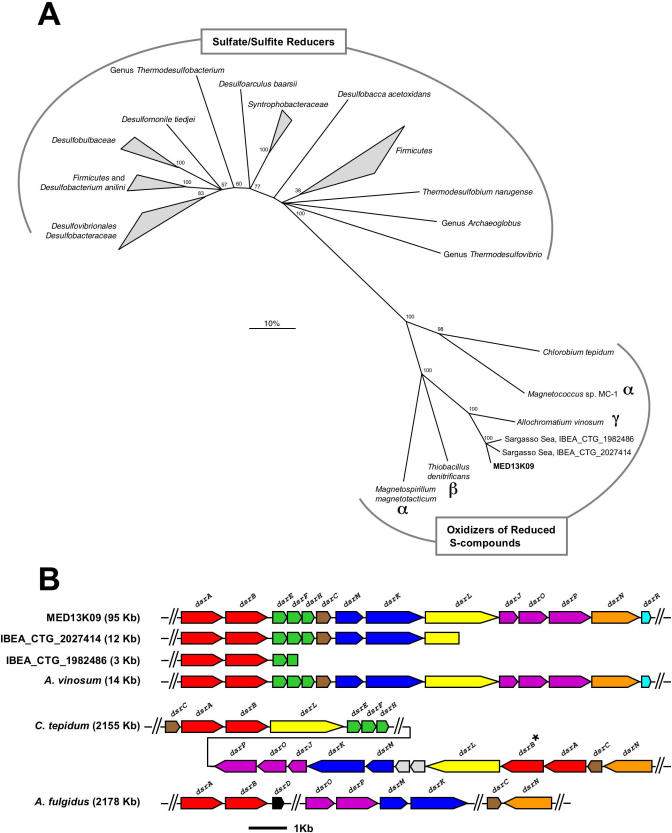
Dsr Operons in PR-Carrying BAC and Sargasso Sea Scaffolds (A) Phylogenetic tree showing the affiliation of DsrAB from MED13K09. Alignment regions of insertions and deletions were omitted in DsrAB amino acid sequence analyses. Polytomic nodes connect branches for which a relative order could not be determined unambiguously by using distance-matrix (FITCH with the Dayhoff PAM matrix, global rearrangements, and randomized input order of species), maximum-parsimony, and maximum-likelihood (with JTT-f as the amino acid replacement model) methods. Maximum-parsimony bootstrap values (%) are indicated at each node (1,000 re-samplings). The bar represents 10% sequence divergence as estimated from distance-matrix analysis. α, *Alphaproteobacteria*; β, *Betaproteobacteria*; γ, *Gammaproteobacteria*. In total, nine Sargasso Sea shotgun clones contained complete (IBEA_CTG_1982486, AACY01045584; IBEA_CTG_2027414, AACY01063972) or partial (IBEA_CTG_UAAO864TF, AACY01493489; IBEA_CTG_SSBMN57TR, AACY01327066; IBEA_CTG_SKBEW15TR, AACY01199346; IBEA_CTG_2002781, AACY01059482; IBEA_CTG_1960714, AACY01122073; IBEA_CTG_2018072, AACY01005285; IBEA_CTG_UAAYT68TR, AACY01523913) *dsrAB* sequences that formed a monophyletic cluster with MED13K09 and *A. vinosum*. Whole-genome shotgun sequence data for *Thiobacillus denitrificans*, *Magnetospirillum magnetotacticum*, and *Magnetococcus* sp. MC-1 were produced by the US Department of Energy Joint Genome Institute (http://www.jgi.doe.gov/). The yet-uncompleted genome sequence of *T. denitrificans* contains a frame shift in *dsrB*. Dissimilatory (bi)sulfite reductase sequences of sulfate-/sulfite reducers were taken from Wagner et al.[40], Klein et al.[41], and Zverlov et al. [42]]. (B) Organization of the *dsr* operons on MED13K09, Sargasso Sea shotgun clones IBEA_CTG_2027414 and IBEA_CTG_1982486, and in *A. vinosum*, *Chlorobium tepidum* TLS, and the sulfate-reducer *Archaeoglobus fulgidus*. Asterisk indicates an authentic frame shift in the second copy of *dsrB* in the genome of *C. tepidum*.

Several interesting operons providing new insights into the metabolisms of PR-encoding microorganisms were linked to PR genes or found on PR-containing BACs. On clone MED13K09, an entire *dsr* operon containing the genes for both subunits of a reverse siroheme sulfite reductase *(dsrAB),* typically used by chemotrophic or anaerobic phototrophic bacteria for exploiting reduced sulfur compounds as electron donor [16,17], was found. The reverse sulfite reductase encoded on this BAC clone forms a highly supported monophyletic cluster with nine reverse sulfite reductases for which genes (or gene fragments) were retrieved from the Sargasso Sea shotgun library [9] and with the respective enzyme of the anaerobic phototrophic purple sulfur bacterium *Allochromatium vinosum* [18] (Figure 3A), a member of the *Gammaproteobacteria*. This grouping is further supported by a highly conserved gene order of other *dsr* genes on the genome fragments (Figure 3B). Furthermore, some but not all phylogenetic analyses of three ribosomal proteins encoded on the genome fragment from BAC clone MED13K09 also suggest that the organism is a deep-branching gammaproteobacterium (Figure S1).

Since we could demonstrate that BAC MED13K09 is not a chimera (Figure S2), the close relationship of the reverse sulfite reductase from the PR-carrying MED13K09 clone with the enzyme of the gammaproteobacterium *A. vinosum* might suggest the existence of a novel anoxygenic phototroph exploiting light for energy generation not only by its bacteriochlorophyll-containing photosystem but also by PR. Alternatively, these genes might originate from a novel chemotrophic oxidizer of reduced sulfur compounds. In this context, it is interesting to note that some anoxygenic phototrophs [19] closely related to *A. vinosum* as well as thiobacilli [20], which both possess *dsrAB* genes [17] (Figure 3), are capable of gaining energy from aerobic oxidation of dimethyl sulfide to sulfate. In contrast to reduced inorganic sulfur compounds, dimethyl sulfide is present in the analyzed oxygenated marine surface waters [21], and PR- and DsrAB-exploiting marine bacteria might thus be involved in degradation of this compound, which plays the key role in the transport of sulfur from oceanic to terrestrial systems [22] and as a precursor for cloud condensation nuclei [23]. Together with the recent finding that SAR11 bacteria consume significant amounts of dimethylsulfoniopropionate [24], an osmoprotectant produced by marine algae and plant halophytes that is degraded by marine bacteria to DMS [25], our results suggest that bacteria exploiting PR phototrophy might be of importance for sulfur cycling in the marine photic zone.

**Figure 4:**
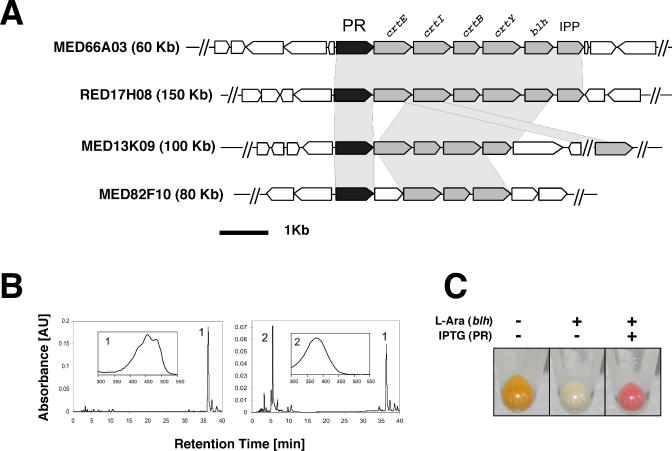
Retinal Biosynthesis Pathways in PR-Carrying BACs (A) Schematic comparison of different carotenoid biosynthesis gene clusters linked to PR genes. ORF marked in gray represent predicted carotenoid biosynthesis genes while PR is marked in black. (B) HPLC separation of the retinoids formed in the β-carotene producing *E. coli* and expressing the Blh protein. Left panel, extract from non-induced cells; right panel, after 60 min of induction (L-arabinose). Insights, absorption spectra of peaks 1 (β-carotene) and 2 (all-*trans* retinal). (C) Color shift due to the cleavage of β-carotene to retinal in *E. coli* cells*.* Color shift from *orange* (β-carotene; non induced) to almost *white* (retinal; L-arabinose induced cells) in β-carotene producing and accumulating *E. coli* cells caused by expression of the *blh* gene and, the same β-carotene producing cells co-expressing the *blh* and a PR gene; color shift to *red* (L-arabinose and IPTG induced cells).

Another interesting genomic feature linked to PR genes was a carotenoid biosynthesis gene cluster found on clones MED66A03, MED13K09, RED17H08, and MED82F10 (Figure 4 and Tables S1–S4). The arrangement of the respective genes was similar, containing the gene order *crtIBY* in all BACs. These genes are predicted to encode for phytoene desaturase, phytoene synthase, and lycopene cyclase, respectively, which catalyze the formation of β-carotene from geranylgeranyl pyrophosphate through phytoene and lycopene intermediates [26]. In addition, the first gene in the carotenoid biosynthesis pathway coding for geranylgeranyl diphosphate synthase *(crtE)* was found in the same operon in MED66A03, RED17H08. MED13K09 carries the *crtE* gene outside the operon approximately 25 kilobases downstream. This suggests that bacteria carrying these operons can synthesize β-carotene. Interestingly, the first reported bacterial gene coding for a homolog of the bacteriorhodopsin-related-protein-like homolog protein (Blh) from the archaeon *Halobacterium* sp. NRC-1 was found in the operons of MED66A03, RED17H08, and MED13K09, leading to the operonal arrangement of *crtEIBY*, *blh* on MED66A03, RED17H08 and *crtIBY*, *blh* on MED13K09. Bacteriorhodopsin-related protein was recently implicated in retinal biosynthesis [27] and was suggested to be the protein converting β-carotene to retinal, similar to the activity of 15,15′-β-carotene dioxygenase from *Drosophila melanogaster* [28]. Although highly speculative, as the identity between the archaeal Blh and the bacterial proteins is only 20%, this may imply that bacteria possessing PR apoproteins also carry the ability to synthesize the retinal chromophore and to potentially form functional PR holoproteins. Indeed, expression of the Blh homolog in β-carotene-producing *E. coli* cells resulted in the loss of the yellow color of these cells (Figure 4). When checked via HPLC, a clear all-*trans* retinal signal was seen only in cells expressing the Blh gene. Moreover, co-expression of the bacterial Blh homolog on a β-carotene-producing and PR-expressing *E. coli* background produced red-colored cells, indicating that the β-carotene is cleaved by the Blh homolog to retinal, which enters the membrane to form an active PR. The β-carotene cleaving enzyme Blh is the first one of its kind found in *bacteria*. The recently reported retinal biosynthetic enzyme from *Synechocystis* PCC 6803 [29] cleaves apo-carotenoids only (i.e., single-ringed carotenes), while the bacterial Blh cleaves β-carotene. In addition, a predicted gene encoding for isopentenyl diphosphate isomerase was found in the carotenoid biosynthetic operons containing the *blh* gene. This protein was shown to enhance isoprenoid biosynthesis when expressed in *E. coli* cells [30].

By taking advantage of large insert environmental BAC libraries and heterologous expression assays, we were able to show that PR-carrying bacteria are an important component of the microbial communities in the photic zone of the Mediterranean Sea and Red Sea, and that several phylogenetically diverse PR genes encode functional light-driven proton pumps. Furthermore, we revealed previously unrecognized links between PR genes and different and partly unexpected metabolic traits and thus gained novel insights into the biology of some uncultured PR-carrying bacteria. Some of these PR-carrying bacteria are apparently energy scavengers, ideally adapted to oligotrophic marine surface waters by exploiting not only light but possibly also some reduced organic sulfur compounds for energy generation.

## Materials and Methods

### BAC library construction

BAC libraries were constructed from plankton samples collected in the Red Sea or from 12-m water collected on a transect from Haifa to Cyprus (33°25′N, 33°56'E to 32°54'N, 34°44'E). BAC library construction was carried out as previously described [10] with minor modifications (for more details, see http://www.tigr.org/tdb/MBMO/MBMO.shtml). The approximately 800 l of pre-filtered waters (Whatman GF/A filter) (Middlesex, United Kingdom) were collected and filtered by tangential flow filtration with a Millipore (Billerica, Massachusetts, United States) Pellicon-2 unit equipped with a C screen Biomax (Martinsried, Germany) 30 polysulfone membrane cartridge (30,000 MW cutoff and 0.5 m^2^ cassette). Bacterioplankton cells were collected by centrifugation of the retentate (4°C, 38,900 × *g*, 1 h). The bacterioplankton pellet was embedded in agarose plugs, and DNA was extracted and cloned into the pBACindigo356 vector. The library consists of 10,560 clones with an average insert size of 80 Kb with a coverage of approximately 850 Mb.

BAC clones chosen for sequencing (black squares in Figure 1) were first screened by low coverage and then followed by a deeper coverage (BAC RED22E04: [estimated size 40 Kb] [72 reads, genomic coverage by trimmed read bases of quality ≥ 20 is 1.06×], 13 contigs assembled that sum to 21 Kb; BAC MED42A11: [estimated size 75 Kb] [458 reads, genomic coverage by trimmed read bases of quality ≥ 20 is 5.10×], eight contigs assembled that sum to 14.5 Kb; MED86H08: [estimated size 35Kb] [45 reads, genomic coverage by trimmed read bases of quality ≥ 20 is 1.04×], six contigs assembled summing to 16 Kb; BAC MED18B02: [estimated size 35Kb], 4 contigs assembled that sum to 32.5 Kb, [364 reads, genomic coverage by trimmed read bases of quality ≥ 20 is 6.12×]; BAC MED46A06: [estimated size 70 Kb], 1 contigs assembled that sum to 69.2 Kb [645 reads, genomic coverage by trimmed read bases of quality ≥ 20 is 7.65×]). BAC clones listed in Tables S1–S6 were completely sequenced (BACs MED13K09 [938 reads, genomic coverage by trimmed read bases of quality ≥ 20 is 6.78×], MED66A03 [621 reads, genomic coverage by trimmed read bases of quality ≥ 20 is 7.48×], MED49C08 [342 reads, genomic coverage by trimmed read bases of quality ≥ 20 is 4.14×], MED35C06 [256 reads, genomic coverage by trimmed read bases of quality ≥ 20 is 4.10×] and RED17H08 [1,800 reads, genomic coverage by trimmed read bases of quality ≥ 20 is 9.4×]) or sequenced to approximately 8× coverage (BAC MED82F10 [1,009 reads, genomic coverage by trimmed read bases of quality ≥ 20 is 8.81×; 11 contigs were assembled that sum to 82 Kb, BAC MED82F10 insert size is approximately 85 Kb]). All BAC insert size estimations were based on NotI restriction digest and CHEF pulse field gel electrophoresis separation, 13 h at 12 °C in 0.5 × TBE buffer at 6 V cm^−1^ with 5–15-s pulses. Sequencing of subclone shotgun libraries from the different BACs were performed by Macrogen (Seoul, Korea). Sequencing reads were trimmed of vector using SEQUENCHER 4.1.2 software (Gene Codes, Ann Arbor, Michigan, United States) and further trimmed manually after chromatograms were inspected for ambiguities. Trimmed and edited sequences were assembled using SEQUENCHER 4.1.2. Additional ambiguities were corrected by examining chromatograms manually, and a consensus for base calling was determined. Gap filling was performed by primer walking or PCR amplification using gap end sequences as primers and sequencing of the PCR product. Direct BAC end-sequencing reads were used to confirm the assembly of the different BACs.

### PR detection in BAC libraries

PR presence in the BAC libraries was detected via Southern hybridizations or by multiplex PCR [31]. Southern hybridizations were performed essentially according to [8] using a mix of different PR genes (eBAC31A08, PalE6, medA15r8ex4, and NA13r8_9) as probes. PR detection via PCR was achieved by using degenerated primers 5′-MGNTAYATHGAYTGGYT-3′, 5′-WWNMGNTAYGTNGAYTGG-3′, and 5′- GGRTADATN GCCCANCC-3′ targeting the conserved RYIDWL, [L/Y/F/Y]RYVDW and GWAIYP regions, respectively

### PR expression and activity

PR representatives (MED13K09, MED18B02, MED46A06, MED49C08, MED66A3, and MED82F10) from different PR groups were expressed in *E. coli* cells using the pBAD expression system [1], and their light-driven proton-pumping activity was measured as previously described [32].

### β-carotene dioxygenase activity

XLI-Blue *E. coli* cells transformed with pBCAR [33] with the *crtE, crtB, crtI,* and *crtY* genes for β-carotene biosynthesis from *Erwinia herbicola,* pGB-Ipi carrying *ipi* (IPP isomerase to DMAPP) from *Haematococcus* *pluvialis* [34] and plasmid pBAD-Blh carrying the *blh* gene under the arabinose promoter were grown overnight at 37 °C in the dark to early stationary phase. Bacteria were harvested from samples of 10 ml of culture for carotenoids and retinoids analysis at time 0, 1, 2, and 6 h after addition of 0.1% (w/v) L-arabinose. Uninduced cells were harvested at 6 h of growth. Cells were resuspended in 200 μl of 6M formaldehyde and incubated for 2 min at 37 °C. Two ml of dichloromethane were added, and carotenoids and retinoids were extracted twice with 4 ml of hexane. The solvent was dried under a stream of nitrogen and the carotenoids dissolved in 75 μl hexan:ethanol 99.5:0.5 to be injected to the HPLC. Carotenoids and retinoids were separated by HPLC using a Waters (Milford, Massachusetts, United States) system and a Spherisorb ODS2 C18 (5 μm, 4.6 × 250 mm) reversed-phase column. Samples of 25 μl were injected to a Waters 600 pump. A gradient of acetonitrile:water (9:1) containing 0.1% (w/v) ammonium acetate (A) and ethylacetate (B), at a constant flow rate of 1.6 ml/min was used as follows: 100% A during the first 15 min; 100% to 80% A during 8 min; 80% to 65% A during 4 min, followed by 65% to 45% A during 14 min and a final segment at 100% B. Light absorption peaks were detected in the range of 200–600 nm using a Waters 996 photodiode array detector. Carotenoids and retinoids were identified by their absorption spectra and characteristic retention time. Pure all-*trans*-retinal and pure β-carotene were used as standards.

Alternatively, for the co-expression with the PR, *Blh* homolog from MED66A03 was amplified using the BLH66A3fwd 5′- ACCATGGGTGGCTTGATGTTAATTGATTGGTG-3′ and BLH66A3rev 5′- ATTTTTGATTTTAATTCTGGAAGAGTGTGGTC-3′ primers, cloned into the pBAD-TOPO (Invitrogen, Carlsbad, California, United States) expression vector and transformed into β-carotene accumulating *E. coli* cells carrying plasmid p ACCAR16ΔcrtX with the *crtE, crtB, crtI,* and *crtY* genes for β-carotene biosynthesis from *E. herbicola* [26]. For the co-expression with the PR gene, the *blh* gene was cut out using NcoI and PmeI restriction enzymes and cloned into a pBAD-TOPO-derived plasmid carrying the 31A08 PR gene under the control of the lacUV-promoter [K.-H. Jung, V. D. Trivedi, E. N. Spudich, J. L. Spudich, unpublished data]. β-carotene-accumulating *E. coli* cells were grown with L-arabinose and IPTG to induce the *blh* and PR genes, respectively.

## Supporting Information

Figure S1 Phylogenetic Analysis of Ribosomal Proteins L21, L27, and S20 from BAC Clone MED13K09 Ribosomal protein L31, which is also present on BAC clone MED13K09, was excluded from the analysis because lateral gene transfer of this protein has been reported [35]. The dataset consisted of 93 reference organisms, which represent all bacterial genera for which whole genome sequences have been reported. A concatenated dataset and a 30% amino acid sequence conservation filter (234 alignment positions) was used for phylogeny inference. Polytomic nodes connect branches for which a relative order could not be determined unambiguously by using distance-matrix, maximum-parsimony, and maximum-likelihood methods. In contrast to the consensus tree, trees inferred by distance matrix (DM) and maximum-likelihood (ML) methods do support a clustering of MED13K09 proteins with the *Gamma*-/*Betaproteobacteria* (see insets).
(690 KB JPG).

Figure S2 BAC Clone MED13K09 Is Not a Chimera Schematic illustration showing that BAC clone MED13K09 is not a chimera and that the *dsr* genes identified are linked to the PR gene on the genome of the respective unknown marine bacterium. In addition to BAC clone MED13K09, a partially overlapping BAC clone (MED47G02) was detected by BAC end sequencing. This clone does also carry *dsrA* (>100% identity on DNA level) as demonstrated by PCR amplification and sequencing. Specific primer sets were designed and used to amplify overlapping 4-kilobase PCR fragments (shown in red) (using DNA isolated directly from the environment as a template), which demonstrate that the sequence region of MED13K09 identical to the 3′ end of MED47G02 is actually connected to the PR gene. *DsrAB*, *crtE*, PR, and BAC MED47G02 end positions relative to BAC MED13K09 are marked. In addition, a shotgun sequence scaffold from Sargasso Sea carrying *dsr* genes and a PR has been deposited by Venter et al.[9], providing independent evidence for co-occurrence of these genes on bacterial genomes.
(213 KB JPG).

Table S1 List of Genes on BAC Clone MED13K09 This clone contains four genes encoding ribosomal proteins (S20, L27, L21, L31). Based on these proteins, a phylogenetic analysis was performed (see Figure S1). Of the 100 ORFs annotated, 54%, 12%, and 34% were provisionally assigned based on the top BLAST hit to the *Gammaproteobacteria, Alphaproteobacteria,* and other prokaryotes, respectively.
(124 KB DOC).

Table S2 List of Genes on BAC Clone MED66A03 Of the 40 ORFs annotated, 15%, 50%, and 35% were provisionally assigned based on the top BLAST hit to the *Gammaproteobacteria*, *Alphaproteobacteria*, and other prokaryotes, respectively.
(60 KB DOC).

Table S3 List of Genes on BAC Clone RED17H08 Of the 38 ORFs annotated, 16%, 42%, and 42% were provisionally assigned based on the top BLAST hit to the *Gammaproteobacteria,* *Alphaproteobacteria,* and other prokaryotes, respectively.
(62 KB DOC).

Table S4 List of Genes on BAC Clone MED82F10 Of the 18 ORFs annotated, 28%, 22%, and 50% were provisionally assigned based on the top BLAST hit to the *Gammaproteobacteria,* *Alphaproteobacteria,* and other prokaryotes, respectively.
(41 KB DOC).

Table S5 List of Genes on BAC Clone MED49C08 ^a^ORF has highest homology to a protein from the SAR86-related environmental BAC clone EBAC31A08 [1]. Of the 67 ORFs annotated, 60%, 25%, and 15% were provisionally assigned based on the top BLAST hit to the *Gammaproteobacteria, Alphaproteobacteria,* and other prokaryotes, respectively.
(103 KB DOC).

Table S6 List of Genes on BAC Clone MED35C06 ^a^ORF has highest homology to a protein from the SAR86-related environmental BAC clone EBAC31A08 [1]. Of the 39 ORFs annotated, 77%, 13%, and 10% were provisionally assigned based on the top BLAST hit to the *Gammaproteobacteria*, *Alphaproteobacteria*, and other prokaryotes, respectively.
(58 KB DOC).

### Accession Numbers

The GenBank (http://www.ncbi.nlm.nih.gov/Genbank) accession numbers for the genes and gene products discussed in this paper are: *A. vinosum* (U84760); *Archaeoglobus fulgidus* (NC_000917); BAC sequences (BAC MED13K09–DQ068067, BAC MED18B02 [DQ088853–DQ088855], BAC MED35C06–DQ077553, BAC MED42A11 [DQ088869–DQ088876], BAC MED46A06–DQ088847, BAC MED49C08–DQ077554, BAC MED66A03–DQ065755, BAC MED82F10–DQ073796, BAC MED86H08 [DQ088848–DQ088852], BAC RED17H08–DQ068068, BAC RED22E04 [DQ088856–DQ088868]); *Chlorobium tepidum* TLS (NC_002932); *dsr* (IBEA_CTG_1960714, AACY01122073; IBEA_CTG_1982486, AACY01045584; IBEA_CTG_1982486; IBEA_CTG_2002781, AACY01059482; IBEA_CTG_2018072, AACY01005285; IBEA_CTG_2027414, AACY01063972; IBEA_CTG_SKBEW15TR, AACY01199346; IBEA_CTG_SSBMN57TR, AACY01327066; IBEA_CTG_UAAO864TF, AACY01493489; IBEA_CTG_UAAYT68TR, AACY01523913); *Magnetococcus* sp. MC-1 (NZ_AAAN02000009); *Magnetospirillum magnetotacticum* (NZ_AAAP01003703); PR (AY599897–AY599909, CH024977, DQ062244–DQ062293); and *Thiobacillus denitrificans* (NZ_AAFH01000002).

## Abbreviations

BAC: bacterial artificial chromosome
Blh: bacteriorhodopsin-related-protein-like homolog protein
PR: proteorhodopsin
