# Supplementary material for: New Insights into Metabolic Properties of Marine Bacteria Encoding Proteorhodopsins
Source: PLoS Biol. 2005 Jul 19;3(8):e273. doi: 10.1371/journal.pbio.0030273 (PMC1175822; doi:10.1371/journal.pbio.0030273)
Supplement: Supplementary file 1 [file pbio.0030273.st001.doc]

Table S1. List of genes on BAC clone MED13K09. This clone contains four genes encoding ribosomal proteins (S20, L27, L21, L31). Based on these proteins a phylogenetic analysis was performed (see Figure S1).

| ORF | | | | Closest BLASTp hit | | | |
| --- | --- | --- | --- | --- | --- | --- | --- |
| Number | Start | Stop | Dir. | Description | Protein_ID | E-value | Taxonomy of best blast hit |
| 1 | 326 | 1618 | + | Organic solvent tolerance precursor XF0837 | B82756 | 3,00E-36 | Gammaproteobacteria |
| 2 | 1509 | 2831 | + | Survival protein surA (surA) | AAN56623 | 2,00E-38 | Gammaproteobacteria |
| 3 | 2831 | 3808 | + | 4-hydroxythreonine-4-phosphate dehydrogenase (4-(phosphohydroxy)-L-threonine dehydrogenase) (pdxA) | CAE12905 | 1,00E-67 | Gammaproteobacteria |
| 4 | 4140 | 4562 | + | Dimethyladenosine transferase NMA0902 | F81936 | 1,00E-22 | Betaproteobacteria |
| 5 | 4549 | 5214 | + | Hypothetical protein mlr1316 | BAB48719 | 2,00E-09 | Alphaproteobacteria |
| 6 | 5751 | 5245 | - | Hypothetical protein XCC2039 | AAM41328 | 6,00E-07 | Gammaproteobacteria |
| 7 | 5998 | 6465 | + | Bacterioferritin comigratory protein XF0961 | AAF83771 | 2,00E-35 | Gammaproteobacteria |
| 8 | 6692 | 7033 | + | Protein kinase C inhibitor chPKCI | BAA93454 | 7,00E-27 | Eukarya |
| 9 | 7801 | 7040 | - | CG7362-PA CG7362 | AAF55096 | 4,00E-04 | Eukarya |
| 10 | 7847 | 8629 | + | Sterol desaturase VV1721 | BAC94485 | 4,00E-20 | Gammaproteobacteria |
| 11 | 8803 | 9162 | + | No BLASTp hit | - | - | - |
| 12 | 10424 | 9234 | - | Xylene monooxygenase hydroxylase component (xylM) | AAB70825 | 2,00E-29 | Gammaproteobacteria |
| 13 | 10836 | 10597 | - | Ribosomal protein L31 | AAO10246 | 3,00E-18 | Gammaproteobacteria |
| 14 | 10899 | 11642 | + | 3',5'-cyclic-nucleotide phosphodiesterase (EC 3.1.4.17) (cpdA) | AAC22058 | 1,00E-23 | Gammaproteobacteria |
| 15 | 12938 | 11817 | - | AmtB1 (amtB1) | AAL83554 | 5,00E-97 | Gammaproteobacteria |
| 16 | 15171 | 14215 | - | Octaprenyl-diphosphate synthase (octaprenyl pyrophosphate synthetase) (ispB) | CAE16915 | 2,00E-51 | Gammaproteobacteria |
| 17 | 13110 | 14216 | + | Hypothetical protein | AAC06471 | 4,00E-27 | Aquificae |
| 18 | 15268 | 15573 | + | Ribosomal protein L21 (rplU) | AAM24169 | 2,00E-15 | Firmicutes (Clostridia) |
| 19 | 15578 | 15838 | + | Ribosomal protein L27 (rpmA) | CAE16913 | 1,00E-22 | Gammaproteobacteria |
| 20 | 15857 | 16870 | + | GTP-binding protein, GTP1/Obg family | AAO54341 | 8,00E-78 | Gammaproteobacteria |
| 21 | 18246 | 17944 | - | Ribosomal protein S20 (rpsT) | AAO54344 | 9,00E-11 | Gammaproteobacteria |
| 22 | 16848 | 17951 | + | Glutamate 5-kinase (proB) | AAM24501 | 2,00E-67 | Firmicutes (Clostridia) |
| 23 | 18282 | 19850 | + | Virulence factor MviN (mviN) | B81217 | 6,00E-88 | Betaproteobacteria |
| 24 | 19850 | 20764 | + | Riboflavin biosynthesis protein (ribF) | AAN50647 | 3,00E-42 | Spirochaetes |
| 25 | 20757 | 23531 | + | Isoleucyl-tRNA synthetase (ileS) | AAO09026 | 0.0 | Gammaproteobacteria |
| 26 | 23516 | 23998 | + | Signal peptidase II (EC 3.4.23.36) | AAL52980 | 6,00E-08 | Alphaproteobacteria |
| 27 | 23988 | 24416 | + | Peptidyl-prolyl cis-trans isomerase, FkbP-type PA4558 | AAG07946 | 7,00E-10 | Gammaproteobacteria |
| 28 | 24406 | 25353 | + | LytB (lytB) | AAD55804 | 5,00E-92 | Gammaproteobacteria |
| 29 | 25717 | 25364 | - | DsrR (dsrR) | AAG13087 | 2,00E-10 | Gammaproteobacteria |
| 30 | 27062 | 25701 | - | DsrN (dsrN) | AAG13086 | 1,00E-90 | Gammaproteobacteria |
| 31 | 28254 | 27082 | - | DsrP (dsrP) | AAG13085 | 2,00E-82 | Gammaproteobacteria |
| 32 | 28979 | 28254 | - | DsrO (dsrO) | AAG13084 | 2,00E-66 | Gammaproteobacteria |
| 33 | 29280 | 28975 | - | DsrJ (dsrJ) | AAG13083 | 2,00E-14 | Gammaproteobacteria |
| 34 | 31428 | 29458 | - | DsrL (dsrL) | AAG13082 | 0.0 | Gammaproteobacteria |
| 35 | 33006 | 31510 | - | DsrK (dsrK) | AAC35401 | 0.0 | Gammaproteobacteria |
| 36 | 33745 | 33011 | - | DsrM (dsrM) | AAC35400 | 6,00E-60 | Gammaproteobacteria |
| 37 | 34105 | 33776 | - | DsrC (dsrC) | AAC35399 | 1,00E-45 | Gammaproteobacteria |
| 38 | 34421 | 34122 | - | DsrH (dsrH) | AAC35398 | 5,00E-22 | Gammaproteobacteria |
| 39 | 34825 | 34424 | - | DsrF (dsrF) | AAM72092 | 2,00E-31 | Chlorobi (Chlorobia) |
| 40 | 35201 | 34812 | - | DsrE (dsrE) | AAC35396 | 2,00E-53 | Gammaproteobacteria |
| 41 | 36299 | 35217 | - | Siroheme sulfite reductase, beta subunit (dsrB) | AAC35395 | 0.0 | Gammaproteobacteria |
| 42 | 37534 | 36302 | - | Siroheme sulfite reductase, alpha subunit (EC 1.8.99.3) (dsrA) | AAC35394 | 0.0 | Gammaproteobacteria |
| 43 | 37688 | 38524 | + | No BLASTp hit | - | - | - |
| 44 | 38727 | 39401 | + | Hypothetical protein LA3189 | AAN50387 | 3,00E-08 | Spirochaetes |
| 45 | 39465 | 39827 | + | DsrC (dsrC) | AAC35399 | 2,00E-17 | Gammaproteobacteria |
| 46 | 40822 | 40262 | - | No BLASTp hit | - | - | - |
| 47 | 40863 | 41459 | + | Hypothetical protein SO1944 | AAN54995 | 4,00E-11 | Gammaproteobacteria |
| 48 | 41459 | 42562 | + | Hypothetical protein sll1634 | BAA17493 | 6,00E-19 | Cyanobacteria |
| 49 | 42562 | 42861 | + | 3-phenylpropionate dioxygenase ferredoxin subunit (digoxigenin ferredoxin subunit) (hcaC) | CAE14499 | 6,00E-10 | Gammaproteobacteria |
| 50 | 42861 | 43307 | + | Hypothetical protein SCE39.30 | CAB40338 | 0.048 | Actinobacteria |
| 51 | 43933 | 43310 | - | Biopolymer transport ExbB-like transmembrane protein RSc2528 RS01053 (exbB2) | CAD16235 | 2,00E-14 | Betaproteobacteria |
| 52 | 45997 | 44006 | - | TonB-dependent receptor (nosA) | AAN53708 | 8,00E-37 | Gammaproteobacteria |
| 53 | 48798 | 46606 | - | Outer membrane protein SMc02721 | CAC46962 | 7,00E-45 | Alphaproteobacteria |
| 54 | 48939 | 49322 | + | Biopolymer transport protein blr3907 | BAC49172 | 2,00E-04 | Alphaproteobacteria |
| 55 | 49309 | 49704 | + | Hypothetical protein glr1388 | BAC89329 | 0.004 | Cyanobacteria |
| 56 | 51644 | 50244 | - | Siroheme synthase PA2611 | AAG05999 | 1,00E-126 | Gammaproteobacteria |
| 57 | 49655 | 50254 | + | Membrane protein YPO3918 | CAC93384 | 3,00E-07 | Gammaproteobacteria |
| 58 | 52086 | 51652 | - | Hypothetical protein PM1611 | AAK03695 | 2,00E-11 | Gammaproteobacteria |
| 59 | 52337 | 52089 | - | Glutaredoxin | AAF85392 | 6,00E-19 | Gammaproteobacteria |
| 60 | 52674 | 52342 | - | Phage repressor (prpA) | AAM08234 | 8,00E-04 | Gammaproteobacteria |
| 61 | 55672 | 52874 | - | PEP carboxylase (ppc) | BAC48220 | 1,00E-137 | Alphaproteobacteria |
| 62 | 56237 | 55767 | - | Nitrogen fixation protein blr7496 | BAC52761 | 1,00E-21 | Alphaproteobacteria |
| 63 | 57582 | 56251 | - | Dihydroorotase | AAK22375 | 1,00E-113 | Alphaproteobacteria |
| 64 | 57637 | 58029 | + | Hypothetical protein | - | 3,00E-08 | Gammaproteobacteria |
| 65 | 58884 | 58042 | - | No BLASTp hit | - | - | - |
| 66 | 59792 | 58890 | - | Invasin-like SivH (sivH) | AAF65573 | 7,00E-04 | Gammaproteobacteria |
| 67 | 60614 | 59844 | - | O-linked GlcNAc transferase | AAF26789 | 2,00E-14 | Eukarya |
| 68 | 62028 | 60610 | - | Hypothetical protein tlr1271 | BAC08823 | 5,00E-23 | Cyanobacteria |
| 69 | 62738 | 62139 | - | Hypothetical protein mll4476 | BAB51125 | 2,00E-25 | Alphaproteobacteria |
| 70 | 62913 | 64550 | + | Hypothetical protein blr6742 | BAC52007 | 4,00E-54 | Alphaproteobacteria |
| 71 | 64540 | 66615 | + | Oxidoreductase RSc2859 | CAD16566 | 1,00E-125 | Betaproteobacteria |
| 72 | 66704 | 67129 | + | Hypothetical protein tlr0189 | BAC07742 | 1,00E-12 | Cyanobacteria |
| 73 | 67794 | 67369 | - | No BLASTp hit |  | - | - |
| 74 | 68615 | 67794 | - | Hypothetical protein | BAB32258 | 2,00E-31 | Eukarya |
| 75 | 69444 | 68623 | - | Dimethylallyltransferase FN1327 | AAL95523 | 4,00E-36 | Firmicutes |
| 76 | 69456 | 70145 | + | Hypothetical protein VC1723 | C82165 | 2,00E-22 | Gammaproteobacteria |
| 77 | 71014 | 70148 | - | Glucose-1-phosphate thymidylyltransferase (rfbA) | AAQ61674 | 3,00E-85 | Betaproteobacteria |
| 78 | 71552 | 72217 | + | Thiamin biosynthesis protein (thiG) XF0783 | AAF83593 | 6,00E-78 | Gammaproteobacteria |
| 79 | 74310 | 72790 | - | High affinity sulfate transporter | AAC07275 | 4,00E-49 | Aquificae |
| 80 | 72369 | 72794 | + | Hypothetical protein SO3367 | AAN56365 | 6,00E-15 | Gammaproteobacteria |
| 81 | 74249 | 75274 | + | Oxidoreductase Tas, aldo/keto reductase family VV0672 | BAC93436 | 1,00E-101 | Gammaproteobacteria |
| 82 | 75979 | 75299 | - | Acyltransferase PA0005 | AAG03395 | 1,00E-24 | Gammaproteobacteria |
| 83 | 76511 | 75954 | - | Histidinol-phosphatase | AAQ59333 | 3,00E-30 | Betaproteobacteria |
| 84 | 77838 | 76501 | - | Glycyl-tRNA synthetase | AAF11606 | 1,00E-148 | Deinococcus-Thermus |
| 85 | 78100 | 79509 | + | Glutamate-ammonia ligase (EC 6.3.1.2) | AAA25312 | 1,00E-177 | Gammaproteobacteria |
| 86 | 79557 | 80636 | + | Nitrogen regulation protein (ntrB) | AAO53897 | 2,00E-49 | Gammaproteobacteria |
| 87 | 80626 | 82026 | + | Nitrogen regulation protein VV0196 | BAC92960 | 1,00E-126 | Gammaproteobacteria |
| 88 | 83028 | 82027 | - | GpsA (gpsA) | AAP56633 | 1,00E-20 | Firmicutes (Mollicutes) |
| 89 | 83465 | 83031 | - | Protein-transport protein (secB) | AAK38717 | 5,00E-22 | Gammaproteobacteria |
| 90 | 83869 | 83465 | - | Secreted protein STY4092 | CAD03291 | 2,00E-13 | Gammaproteobacteria |
| 91 | 84219 | 83881 | - | Transcriptional regulator, ArsR family | AAQ61015 | 6,00E-24 | Betaproteobacteria |
| 92 | 84297 | 85478 | + | Carboxyl-terminal proteinase PA5134 | AAG08519 | 3,00E-84 | Gammaproteobacteria |
| 93 | 85486 | 86490 | + | Hypothetical protein Atu3651 | AAL44463 | 4,00E-08 | Alphaproteobacteria |
| 94 | 86525 | 86839 | + | No BLASTp hit | - | - | - |
| 95 | 87548 | 86973 | - | Hypothetical protein all3194 | BAB74893 | 5,00E-23 | Cyanobacteria |
| 96 | 87938 | 87555 | - | No BLASTp hit | - | - | - |
| 97 | 89878 | 87941 | - | Acetyl-CoA synthase mlr4089 | BAB50829 | 0.0 | Alphaproteobacteria |
| 98 | 90967 | 90287 | - | Brp-like protein, Blh | AAV46921 | 8,00E-6 | Archaea |
| 99 | 92054 | 90936 | - | Lycopene cyclase | CAB53415 | 1,00E-16 | Actinobacteria |
| 100 | 92865 | 92044 | - | Phytoene synthase (crtB) | AAM48647 | 1,00E-27 | - |
| 101 | 94327 | 92861 | - | Phytoene dehydrogenase | AAA24820 | 4,00E-96 | Gammaproteobacteria |
| 102 | 95144 | 94386 | - | Proteorhodopsin | AAO73934 | 4,00E-75 | - |
| 103 | 95523 | 95254 | - | No BLASTp hit | - | - | - |
| 104 | 96340 | 95759 | - | Hypothetical protein | BAC17899 | 7,00E-36 | Actinobacteria |
| 105 | 96293 | 96985 | + | Hypothetical protein | CAE19452 | 1,00E-59 | Cyanobacteria |
| 106 | 97300 | 97010 | - | Hypothetical protein NMB0800 | AAF41213 | 7,00E-15 | Betaproteobacteria |
| 107 | 97665 | 97309 | - | No BLASTp hit | - | - | - |
| 108 | 97826 | 98968 | + | Glycosyl transferase HI1578 | AAC23227 | 0.25 | Gammaproteobacteria |
| 109 | 99558 | 99007 | - | Pfs (pfs) | AAP56766 | 8,00E-09 | Firmicutes (Mollicutes) |

Of the 100 ORFs annotated, 54%, 12%, and 34% were provisionally assigned based on the top BLAST hit to the *Gammaproteobacteria*, *Alphaproteobacteria*, and other prokaryotes, respectively.
