# Supplementary material for: New Insights into Metabolic Properties of Marine Bacteria Encoding Proteorhodopsins
Source: PLoS Biol. 2005 Jul 19;3(8):e273. doi: 10.1371/journal.pbio.0030273 (PMC1175822; doi:10.1371/journal.pbio.0030273)
Supplement: Supplementary file 2 [file pbio.0030273.st002.doc]

Table S2. List of genes on BAC clone MED66A03

| ORF | | | | Closest BLASTp hit | | | |
| --- | --- | --- | --- | --- | --- | --- | --- |
| Number | Start | Stop | Direction | Description | Protein_ID | E-value | Taxonomy of best blast hit |
| 1 | 733 | 266 | - | Hypothetical protein PA2954 | AAG06342 | 7,00E-17 | Gammaproteobacteria |
| 2 | 1011 | 1922 | + | Transmembrane protein SMc02232 | CAC45109 | 1,00E-72 | Alphaproteobacteria |
| 3 | 1922 | 2719 | + | Hypothetical protein mll5621 | BAB52033 | 9,00E-32 | Alphaproteobacteria |
| 4 | 2799 | 3284 | + | Hypothetical protein mll5316 | BAB51789 | 1,00E-25 | Alphaproteobacteria |
| 5 | 3381 | 3887 | + | Hypothetical protein CC1584 | AAK23563 | 4,00E-41 | Alphaproteobacteria |
| 6 | 4798 | 3914 | - | RarD (rarD) | AAN29609 | 2,00E-30 | Alphaproteobacteria |
| 7 | 6099 | 4867 | - | Ferredoxin reductase | AAK25487 | 8,00E-57 | Alphaproteobacteria |
| 8 | 6425 | 6159 | - | No BLASTp hit |  |  |  |
| 9 | 6802 | 7620 | + | Proteorhodopsin | AAO73934 | 5,00E-72 |  |
| 10 | 7682 | 8695 | + | Geranylgeranyl pyrophosphate synthetase (crtE) | AAK40423 | 1,00E-19 | Archaea |
| 11 | 8841 | 10328 | + | Phytoene dehydrogenase (crtI) | BAA94063 | 1,00E-121 | Betaproteobacteria |
| 12 | 10350 | 11306 | + | Phytoene synthase (crtB) | AAF78202 | 6,00E-25 | Alphaproteobacteria |
| 13 | 11306 | 12445 | + | Lycopene cyclase (crtY) | CAA64855 | 3,00E-27 | Actinobacteria |
| 14 | 12439 | 13266 | + | Brp-like protein-like | AAV46921 | 1,00E-07 | Archaea |
| 15 | 13263 | 14318 | + | IPP isomerase | AAA64978 | 5,00E-60 | Gammaproteobacteria |
| 16 | 14786 | 15949 | + | L-lactate dehydrogenase (lctD) | AAM39425 | 1,00E-57 | Gammaproteobacteria |
| 17 | 17958 | 15952 | - | Cold-shock dead-box protein A | AAL44971 | 1,00E-162 | Alphaproteobacteria |
| 18 | 18247 | 18651 | + | Phosphoribosyl-AMP cyclohydrolase | CAE19037 | 9,00E-26 | Cyanobacteria |
| 19 | 19131 | 18673 | - | Molybdopterin converting factor, large subunit PA3916 | AAG07303 | 1,00E-36 | Gammaproteobacteria |
| 20 | 19390 | 19136 | - | Molybdopterin converting factor small subunit (moaD) | BAC52737 | 2,00E-13 | Alphaproteobacteria |
| 21 | 20628 | 19384 | - | Molybdopterin biosynthesis protein (moeA) | BAC47577 | 2,00E-57 | Alphaproteobacteria |
| 22 | 21200 | 20649 | - | Molybdopterin biosynthesis MoeA protein SO4723 | AAN57682 | 5,00E-31 | Gammaproteobacteria |
| 23 | 22210 | 21209 | - | Molybdenum cofactor biosynthesis protein A | AAL42695 | 1,00E-110 | Alphaproteobacteria |
| 24 | 23180 | 22347 | - | Citrate (pro-3S)-lyase (EC 4.1.3.6) | AAL54316 | 1,00E-30 | Alphaproteobacteria |
| 25 | 24031 | 23180 | - | Cytosolic protein blr7247 | BAC52512 | 9,00E-40 | Alphaproteobacteria |
| 26 | 24658 | 24047 | - | Glutathione S-transferase | AAO53992 | 7,00E-25 | Gammaproteobacteria |
| 27 | 24900 | 25934 | + | Hypothetical protein SA2232 | BAB43534 | 7,00E-13 | Firmicutes (Bacilli) |
| 28 | 26510 | 25926 | - | Inner mitochondrial membrane protein Sco1p mlr1532 | BAB48883 | 7,00E-38 | Alphaproteobacteria |
| 29 | 26717 | 27595 | + | Hypothetical protein mlr1349 | BAB48742 | 5,00E-52 | Alphaproteobacteria |
| 30 | 28237 | 27605 | - | No BLASTp hit |  |  |  |
| 31 | 29159 | 28392 | - | D-erythrulose reductase (der) | BAB97210 | 3,00E-39 | Eukarya |
| 32 | 30484 | 29177 | - | Histidinol dehydrogenase mlr6649 | BAB52900 | 1,00E-152 | Alphaproteobacteria |
| 33 | 30990 | 30544 | - | Hypothetical protein | - | 2,00E-39 | Actinobacteria |
| 34 | 31162 | 32127 | + | C4-dicarboxylate transport system C4-dicarboxylate-binding protein OB0243 | BAC12199 | 6,00E-27 | Firmicutes (Bacilli) |
| 35 | 33813 | 32191 | - | Hypothetical protein bll5659 | BAC50924 | 2,00E-91 | Alphaproteobacteria |
| 36 | 33991 | 35037 | + | Malate dehydrogenase (EC 1.1.1.37) | AAB99436 | 4,00E-55 | Alphaproteobacteria |
| 37 | 36420 | 35074 | - | glu-tRNAGln amidotransferase chain A | AAK80617 | 2,00E-41 | Firmicutes (Clostridia) |
| 38 | 36556 | 37821 | + | Chromate transporter (chrA) | BAB04293 | 1,00E-69 | Firmicutes (Bacilli) |
| 39 | 37954 | 40227 | + | Oxidoreducatase RSc1468 RS03839 | CAD15170 | 1,00E-123 | Betaproteobacteria |
| 40 | 43252 | 40250 | - | Hypothetical protein blr6041 | BAC51306 | 0.0 | Alphaproteobacteria |
| 41 | 43316 | 44494 | + | Hypothetical protein bll6039 | BAC51304 | 1,00E-138 | Alphaproteobacteria |
| 42 | 44531 | 44890 | + | 5-oxovalerate dehydrogenase (cpnE) | BAC22647 | 1,00E-20 | Betaproteobacteria |

Of the 40 ORFs annotated, 15%, 50%, and 35% were provisionally assigned based on the top BLAST hit to the *Gammaproteobacteria*, *Alphaproteobacteria*, and other prokaryotes, respectively.
