# Supplementary material for: New Insights into Metabolic Properties of Marine Bacteria Encoding Proteorhodopsins
Source: PLoS Biol. 2005 Jul 19;3(8):e273. doi: 10.1371/journal.pbio.0030273 (PMC1175822; doi:10.1371/journal.pbio.0030273)
Supplement: Supplementary file 3 [file pbio.0030273.st003.doc]

Table S3. List of genes on BAC clone RED17H08

| ORF | | | | Closest BLASTp hit | | | |
| --- | --- | --- | --- | --- | --- | --- | --- |
| Number | Start | Stop | Direction | Description | Protein_ID | E-value | Taxonomy of best blast hit |
| 1 | 12 | 353 | + | High-affinity zinc uptake system protein (znuA) | AAL53419 | 4,00E-36 | Alphaproteobacteria |
| 2 | 599 | 273 | - | No BLASTp hit |  |  |  |
| 3 | 770 | 1816 | + | No BLASTp hit |  |  |  |
| 4 | 1552 | 710 | - | Transcription regulator (IclR family) BH2137 | BAB05856 | 9,00E-20 | Firmicutes (Bacilli) |
| 5 | 1609 | 3201 | + | GMC type oxidoreductase blr2397 | BAC47662 | 1,00E-147 | Alphaproteobacteria |
| 6 | 3201 | 3794 | + | Hypothetical protein AGR_C_3584 | AAK87725 | 7,00E-33 | Alphaproteobacteria |
| 7 | 3946 | 5079 | + | Exported protein | CAE41992 | 3,00E-42 | Betaproteobacteria |
| 8 | 5176 | 7437 | + | Hypothetical protein mll5170 | BAB51665 | 5,00E-50 | Alphaproteobacteria |
| 9 | 7403 | 8737 | + | Histidinol dehydrogenase mlr6649 | BAB52900 | 1,00E-86 | Alphaproteobacteria |
| 10 | 8747 | 9265 | + | Sulfopyruvate decarboxylase, alpha subunit (comD) | AAM01610 | 1,00E-23 | Archaea |
| 11 | 9273 | 9824 | + | Phosphonopyruvate decarboxylase | A64332 | 5,00E-35 | Archaea |
| 12 | 9839 | 10597 | + | Short chain dehydrogenase/reductase MM0185 | AAM29881 | 3,00E-38 | Archaea |
| 13 | 10597 | 11577 | + | Dehydrogenase OB2815 | BAC14771 | 4,00E-31 | Firmicutes (Bacilli) |
| 14 | 11587 | 12459 | + | Hypothetical protein alr2966 | BAB74665 | 2,00E-69 | Cyanobacteria |
| 15 | 12167 | 12889 | + | Hypothetical protein tlr0011 | BAC07564 | 8,00E-11 | Cyanobacteria |
| 16 | 12968 | 14518 | + | Propionyl-CoA carboxylase (EC 6.4.1.3) | AAL51982 | 0.0 | Alphaproteobacteria |
| 17 | 14533 | 16527 | + | Hypothetical protein PccA | AAL44393 | 1,00E-177 | Alphaproteobacteria |
| 18 | 18254 | 16530 | - | Oxidoreductase, GMC family CC0945 | AAK22929 | 1,00E-141 | Alphaproteobacteria |
| 19 | 18261 | 19007 | + | Hypothetical protein blr3870 | BAC49135 | 1,00E-21 | Alphaproteobacteria |
| 20 | 19037 | 19624 | + | DNA-3-methyladenine glycosylase I (tag) | AAQ59619 | 7,00E-48 | Betaproteobacteria |
| 21 | 20097 | 19651 | - | No BLASTp hit |  |  |  |
| 22 | 20403 | 20134 | - | No BLASTp hit |  |  |  |
| 23 | 21302 | 20403 | - | Hemolysin | AAO08926 | 2,00E-26 | Gammaproteobacteria |
| 24 | 21555 | 21214 | - | No BLASTp hit |  |  |  |
| 25 | 21671 | 22552 | + | Proteorhodopsin | AAO73934 | 9,00E-73 |  |
| 26 | 22657 | 23679 | + | Geranylgeranyl pyrophosphate synthase IspA2 (crtE) | AAM25613 | 2,00E-16 | Firmicutes (Clostridia) |
| 27 | 23693 | 25243 | + | Phytoene dehydrogenase (crtI) | BAA94063 | 1,00E-126 | Betaproteobacteria |
| 28 | 25262 | 26179 | + | Phytoene synthase (crtB) | P54975 | 9,00E-27 | Alphaproteobacteria |
| 29 | 25899 | 27281 | + | Lycopene cyclase (crtY) | CAB53415 | 4,00E-29 | Actinobacteria |
| 30 | 27047 | 28138 | + | Brp-like protein | AAG20274 | 0.004 | Archaea |
| 31 | 28165 | 29196 | + | IPP isomerase | AAA64978 | 1,00E-62 | Gammaproteobacteria |
| 32 | 29982 | 29212 | - | Hydroxyacylglutathione hydrolase (glyoxalase II) SMc00708 (gloB) | CAC47243 | 6,00E-68 | Alphaproteobacteria |
| 33 | 30460 | 29975 | - | Lactoylglutathione lyase mll0167 | BAB47809 | 1,00E-59 | Alphaproteobacteria |
| 34 | 30771 | 31991 | + | Hypothetical protein | G96031 | 3,00E-08 | Alphaproteobacteria |
| 35 | 31349 | 30498 | - | Hypothetical protein PA0340 | AAG03729 | 2,00E-29 | Gammaproteobacteria |
| 36 | 31997 | 31578 | - | No BLASTp hit |  |  |  |
| 37 | 32014 | 32385 | + | Hypothetical protein mlr4106 | BAB50844 | 7,00E-18 | Alphaproteobacteria |
| 38 | 32376 | 32747 | + | Hypothetical protein LB348 | AAN51907 | 0.022 | Spirochaetes |
| 39 | 32815 | 33759 | + | Ketopantoate reductase STM2573 | AAL21467 | 4,00E-32 | Gammaproteobacteria |
| 40 | 33425 | 34573 | + | Aldolase PA3430 | AAG06818 | 3,00E-42 | Gammaproteobacteria |
| 41 | 35812 | 34586 | - | Aminotransferase SMc01969 | CAC47082 | 1,00E-113 | Alphaproteobacteria |
| 42 | 37358 | 35769 | - | Phosphomannose isomerase/GDP-mannose pyrophosphorylase (xanB) | AAM39941 | 5,00E-92 | Gammaproteobacteria |
| 43 | 38275 | 38523 | + | Protocatechuate 4,5-dioxygenase alpha subunit (pmdA) | AAM09636 | 9,00E-14 | Betaproteobacteria |
| 44 | 38280 | 37285 | - | Transcription regulatory protein, lysR family BMEII1065 | AAL54307 | 1,00E-27 | Alphaproteobacteria |

Of the 38 ORFs annotated, 16%, 42%, and 42% were provisionally assigned based on the top BLAST hit to the *Gammaproteobacteria*, *Alphaproteobacteria*, and other prokaryotes, respectively.
