# Supplementary material for: New Insights into Metabolic Properties of Marine Bacteria Encoding Proteorhodopsins
Source: PLoS Biol. 2005 Jul 19;3(8):e273. doi: 10.1371/journal.pbio.0030273 (PMC1175822; doi:10.1371/journal.pbio.0030273)
Supplement: Supplementary file 4 [file pbio.0030273.st004.doc]

Table S4. List of genes on BAC clone MED82F10

| ORF | | | | Closest BLASTp hit | | | |
| --- | --- | --- | --- | --- | --- | --- | --- |
| Number | Start | Stop | Direction | Description | Protein_ID | E-value | Taxonomy of best blast hit |
| 1 | 18 | 1676 | + | Hypothetical protein glnD | AAL41368 | 2,00E-49 | Alphaproteobacteria |
| 2 | 1676 | 1996 | + | No BLASTp hit |  |  |  |
| 3 | 2051 | 3052 | + | Tryptophanyl-tRNA synthetase (trpS) | AAL86730 | 1,00E-104 | Alphaproteobacteria |
| 4 | 3103 | 3657 | + | Hypothetical protein mll5315 | BAB51788 | 7,00E-54 | Alphaproteobacteria |
| 5 | 3635 | 4483 | + | Bax protein VV1683 | BAC94447 | 5,00E-24 | Gammaproteobacteria |
| 6 | 5424 | 6104 | + | Hypothetical protein YeaZ (yeaZ) | AAM67869 | 7,00E-04 | Gammaproteobacteria |
| 7 | 5518 | 4490 | - | Polysaccharide deacetylase family protein SO4616 | AAN57576 | 1,00E-22 | Gammaproteobacteria |
| 8 | 6573 | 6112 | - | Peptide methionine sulfoxide reductase | AAB85041 | 2,00E-35 | Archaea |
| 9 | 7702 | 6794 | - | Hypothetical senescence marker protein ST2555 | BAB67667 | 6,00E-28 | Archaea |
| 10 | 9133 | 7808 | - | O-acetylhomoserine sulfhydrylase (metY) | AAN49261 | 1,00E-116 | Spirochaetes |
| 11 | 9190 | 9657 | + | No BLASTp hit |  |  |  |
| 12 | 9651 | 10052 | + | Hypothetical protein | CAA28437 | 0.11 | Eukarya |
| 13 | 10542 | 10069 | - | Hypothetical protein VCA0237 | AAF96148 | 2,00E-38 | Gammaproteobacteria |
| 14 | 11463 | 10567 | - | Geranyltranstransferase (crtE) | CAC99441 | 8,00E-38 | Firmicutes (Bacilli) |
| 15 | 11844 | 12593 | + | Proteorhodopsin | AAO73934 | 7,00E-73 |  |
| 16 | 12665 | 13099 | + | No BLASTp hit |  |  |  |
| 17 | 13286 | 14782 | + | Phytoene dehydrogenase (crtI) | AAL02000 | 1,00E-116 | Alphaproteobacteria |
| 18 | 14787 | 15653 | + | Phytoene desaturase CT1386 (crtB) | AAM72615 | 4,00E-25 | Chlorobi (Chlorobia) |
| 19 | 15643 | 16713 | + | Lycopene cyclase (crtY) | CAB53415 | 7,00E-11 | Actinobacteria |
| 20 | 16718 | 17242 | + | No BLASTp hit |  |  |  |
| 21 | 17181 | 17531 | + | No BLASTp hit |  |  |  |
| 22 | 18465 | 17530 | - | LytB (lytB) | AAK03748 | 6,00E-72 | Gammaproteobacteria |
| 23 | 19226 | 18504 | - | pyruvate carboxylase pycA [imported] - Bacillus halodurans (strain C-125) | BAB06344 | 6,00E-53 | Firmicutes (Bacilli) |

Of the 18 ORFs annotated, 28%, 22%, and 50% were provisionally assigned based on the top BLAST hit to the *Gammaproteobacteria*, *Alphaproteobacteria*, and other prokaryotes, respectively.
