# Supplementary material for: New Insights into Metabolic Properties of Marine Bacteria Encoding Proteorhodopsins
Source: PLoS Biol. 2005 Jul 19;3(8):e273. doi: 10.1371/journal.pbio.0030273 (PMC1175822; doi:10.1371/journal.pbio.0030273)
Supplement: Supplementary file 5 [file pbio.0030273.st005.doc]

Table S5. List of genes on BAC clone MED49C08

| ORF | | | | Closest BLASTp hit | | | |
| --- | --- | --- | --- | --- | --- | --- | --- |
| Number | Start | Stop | Direction | Description | Protein_ID | E-value | Taxonomy of best blast hit |
| 1 | 872 |  | - | Two-component sensor histidine kinase (phoR) | CAD00578 | 5,00E-14 | Firmicutes (Bacilli) |
| 2 | 1573 | 887 | - | Response regulator (CheY-like receiver domain and DNA-binding HTH domain) | AAK79666 | 6,00E-53 | Firmicutes (Clostridia) |
| 3 | 2616 | 1639 | - | Hypothetical protein RSc2084 RS03649 | CAD15791 | 2,00E-73 | Betaproteobacteria |
| 4 | 3476 | 2616 | - | Ser/Thr protein phosphatase family protein | AAO55319 | 2,00E-78 | Gammaproteobacteria |
| 5 | 3502 | 4104 | + | No BLASTp hit |  |  |  |
| 6 | 4573 | 4097 | - | Hypothetical protein mll7959 | BAB53622 | 1,00E-13 | Alphaproteobacteria |
| 7 | 5426 | 4578 | - | Hypothetical protein blr5285 | BAC50550 | 1,00E-47 | Alphaproteobacteria |
| 8 | 6097 | 5453 | - | 3-hydroxyacyl-CoA dehydrogenase OB2673 | BAC14629 | 1,00E-61 | Firmicutes (Bacilli) |
| 9 | 6362 | 7150 | + | Enoyl CoA hydratase (fadB) | BAC48211 | 3,00E-74 | Alphaproteobacteria |
| 10 | 7154 | 7924 | + | Hypothetical protein bll5442 | BAC50707 | 2,00E-75 | Alphaproteobacteria |
| 11 | 7930 | 8727 | + | Hypothetical protein bll0552 | BAC45817 | 2,00E-53 | Alphaproteobacteria |
| 12 | 8734 | 10446 | + | Long chain fatty acid CoA ligase | BAB07805 | 1,00E-103 | Alphaproteobacteria |
| 13 ^a^ | 10451 | 11245 | + | Oxidoreductase | AAG10472 | 3,00E-86 | Gammaproteobacteria |
| 14 | 11587 | 12210 | + | Hypothetical protein (ywaF) | CAA36722 | 9,00E-07 | Firmicutes (Bacilli) |
| 15 | 11609 | 11232 | - | No BLASTp hit |  |  |  |
| 16 | 12307 | 12669 | + | No BLASTp hit |  |  |  |
| 17 | 12769 | 13104 | + | Peptidyl-dipeptidase Dcp (dcp-1) | AAN56143 | 1,00E-15 | Gammaproteobacteria |
| 18 | 13241 | 14857 | + | Peptidyl-dipeptidase A SMc04403 (dcp) | CAC47887 | 1,00E-130 | Alphaproteobacteria |
| 19 | 14877 | 16640 | + | Very-long-chain acyl-CoA synthetase | BAA23644 | 6,00E-98 | Eukarya |
| 20 | 17152 | 17982 | + | Hypothetical protein CC1171 | AAK23155 | 3,00E-28 | Alphaproteobacteria |
| 21 | 18307 | 17900 | - | No BLASTp hit |  |  |  |
| 22 | 18342 | 19079 | + | Cystathionine beta-synthase (cysB2) | AAQ61059 | 4,00E-71 | Betaproteobacteria |
| 23 | 19082 | 19414 | + | Cystathionine gamma-lyase-like protein (metB) | AAM38445 | 9,00E-25 | Gammaproteobacteria |
| 24 | 19302 | 20087 | + | Cystathionine gamma-lyase mll4503 | BAB51144 | 5,00E-72 | Alphaproteobacteria |
| 25 | 20597 | 20244 | - | Formyltetrahydrofolate deformylase RSc1873 RS03433 (purU1) | CAD15575 | 2,00E-37 | Betaproteobacteria |
| 26 | 21099 | 20650 | - | PurU-like protein (fdxE) | CAA72163 | 6,00E-18 | Alphaproteobacteria |
| 27 | 21272 | 22381 | + | FadE17 | CAB06527 | 1,00E-52 | Actinobacteria |
| 28 | 22384 | 23475 | + | Hypothetical protein blr1022 | BAC46287 | 3,00E-35 | Alphaproteobacteria |
| 29 | 24486 | 23485 | - | Hypothetical protein blr7937 | BAC53202 | 2,00E-63 | Alphaproteobacteria |
| 30 | 26468 | 24489 | - | Malate synthase (EC 4.1.3.2) | AAL51561 | 0.0 | Alphaproteobacteria |
| 31 | 26680 | 27219 | + | Succinyl-diaminopimelate desuccinylase (dapE) | AAQ59131 | 6,00E-29 | Betaproteobacteria |
| 32 | 26733 | 26452 | - | Malate synthase (EC 4.1.3.2) | AAA68074 | 3,00E-04 | Actinobacteria |
| 33 | 27158 | 27769 | + | Succinyl-diaminopimelate desuccinylase (dapE) | AAM36303 | 2,00E-49 | Gammaproteobacteria |
| 34 | 27919 | 28557 | + | Alternative oxidase AOX1c | BAA22635 | 2,00E-57 | Eukarya |
| 35 | 28791 | 29705 | + | Regulator protein AsfR (asfR) | AAD31779 | 2,00E-11 | Gammaproteobacteria |
| 36 | 30112 | 29720 | - | No BLASTp hit |  |  |  |
| 37 | 30128 | 30862 | + | Hypothetical protein | CAE19460 | 4,00E-08 | Cyanobacteria |
| 38 | 31616 | 30912 | - | Proteorhodopsin | AAO73934 | 4,00E-63 |  |
| 39 | 33064 | 31697 | - | Damage-inducible protein PAB0243 | CAB49288 | 7,00E-50 | Archaea |
| 40 | 33571 | 33071 | - | Protein disulfide-isomerase (EC 5.3.4.1) XF1834 | AAF84640 | 2,00E-12 | Gammaproteobacteria |
| 41 | 34568 | 33564 | - | Hypothetical protein CC1507 | AAK23486 | 5,00E-52 | Alphaproteobacteria |
| 42 | 34736 | 35131 | + | No BLASTp hit |  |  |  |
| 43 | 35131 | 35520 | + | No BLASTp hit |  |  |  |
| 44 ^a^ | 37565 | 35541 | - | TonB-dependent receptor | AAQ62389 | 1,00E-162 | Gammaproteobacteria |
| 45 ^a^ | 38113 | 37751 | - | TonB-dependent receptor | AAQ62389 | 6,00E-19 | Gammaproteobacteria |
| 46 | 38531 | 38151 | - | Hypothetical protein | AAK01512 | 1,00E-07 | Gammaproteobacteria |
| 47 | 39039 | 38509 | - | Hypothetical protein | AAK01512 | 4,00E-19 | Gammaproteobacteria |
| 48 | 40791 | 39559 | - | AmtB2 (amtB2) | AAL83555 | 2,00E-97 | Gammaproteobacteria |
| 49 | 41103 | 40810 | - | Nitrogen regulatory protein p-II | AAK87538 | 2,00E-22 | Alphaproteobacteria |
| 50 | 42137 | 41421 | - | RNA methyltransferase, TrmH family, group 1 | AAO54941 | 3,00E-41 | Gammaproteobacteria |
| 51 | 42405 | 42881 | + | Extragenic suppressor protein SuhB PA3818 | AAG07205 | 2,00E-38 | Gammaproteobacteria |
| 52 | 43421 | 42987 | - | Protein-export membrane protein SecF VC0744 | AAF93909 | 2,00E-28 | Gammaproteobacteria |
| 53 | 43897 | 43409 | - | Preprotein translocase subunit SecF VV0749 | BAC93513 | 9,00E-23 | Gammaproteobacteria |
| 54 | 44190 | 43891 | - | Secretion protein SecD PA3821 | AAG07208 | 2,00E-28 | Gammaproteobacteria |
| 55 | 45536 | 44169 | - | Secretion protein SecD PA3821 | AAG07208 | 1,00E-107 | Gammaproteobacteria |
| 56 | 46046 | 45729 | - | Hypothetical protein PA3822 | AAG07209 | 2,00E-14 | Gammaproteobacteria |
| 57 | 46200 | 47663 | + | Glycerol-3-phosphate acyltransferase PA3673 | AAG07061 | 2,00E-91 | Gammaproteobacteria |
| 58 | 47620 | 47931 | + | Glycerol-3-phosphate acyltransferase VC0093 | AAF93271 | 8,00E-08 | Gammaproteobacteria |
| 59 | 48141 | 48638 | + | Glycerol-3-phosphate acyltransferase PA3673 | AAG07061 | 2,00E-13 | Gammaproteobacteria |
| 60 | 49514 | 49266 | - | Hypothetical protein (yhiQ) | AAM68101 | 0.004 | Gammaproteobacteria |
| 61 | 50214 | 49963 | - | Undecaprenol kinase SO1293 | AAN54360 | 2,00E-07 | Gammaproteobacteria |
| 62 | 50740 | 50207 | - | Undecaprenol kinase SO1293 | AAN54360 | 3,00E-29 | Gammaproteobacteria |
| 63 | 51403 | 50750 | - | Hypothetical protein (yeaZ) | AAM67869 | 3,00E-16 | Gammaproteobacteria |
| 64 | 51894 | 51403 | - | Adenylate kinase (adk) | BAA75818 | 3,00E-45 | Gammaproteobacteria |
| 65 | 52464 | 52054 | - | Phosphopantetheinyl transferase (holo-ACP synthase) (acpS) | AAM25330 | 1,00E-12 | Firmicutes (Clostridia) |
| 66 | 52787 | 52476 | - | DNA repair/recombination protein (recO) | AAA69692 | 2,00E-04 | Gammaproteobacteria |
| 67 | 54028 | 53129 | - | Era (era) | AAK02144 | 3,00E-68 | Gammaproteobacteria |
| 68 | 54416 | 54024 | - | Ribonuclease III (rnc) | AAM67808 | 5,00E-09 | Gammaproteobacteria |
| 69 | 55180 | 54674 | - | Signal peptidase I (EC 3.4.21.89) | CAA39839 | 5,00E-17 | Gammaproteobacteria |
| 70 | 55652 | 55068 | - | Signal peptidase I (lepB) | AAM36194 | 8,00E-24 | Gammaproteobacteria |
| 71 | 56335 | 55655 | - | GTP-binding protein LepA PA0767 | AAG04156 | 1,00E-76 | Gammaproteobacteria |
| 72 | 57435 | 56329 | - | GTP-binding protein LepA (lepA) | AAO57676 | 1,00E-126 | Gammaproteobacteria |
| 73 | 58891 | 57476 | - | MucD (mucD) | AAK11276 | 1,00E-103 | Gammaproteobacteria |
| 74 | 59594 | 58896 | - | No BLASTp hit |  |  |  |
| 75 | 60165 | 59587 | - | DNA-directed RNA polymerase specialized sigma subunit VV2838 | BAC95602 | 9,00E-37 | Gammaproteobacteria |
| 76 | 60455 | 61132 | + | Hypothetical protein HI0466 | AAC22125 | 1,00E-14 | Gammaproteobacteria |
| 77 | 61804 | 61130 | - | Uracil-DNA glycosylase PA0750 | AAG04139 | 1,00E-60 | Gammaproteobacteria |
| 78 | 62140 | 63228 | + | Oxidoreductase PA0840 | AAG04229 | 1,00E-102 | Gammaproteobacteria |
| 79 | 63991 | 63236 | - | Oxidoreductase bll0137 | BAC45402 | 5,00E-47 | Alphaproteobacteria |
| 80 | 65171 | 63987 | - | D-isomer specific 2-hydroxyacid dehydrogenase family protein SAG1566 | AAN00432 | 5,00E-84 | Firmicutes (Bacilli) |
| 81 | 65235 | 65489 | + | Ornithine carbamoyltransferase (EC 2.1.3.3) Cj0994c | CAB73250 | 6,00E-15 | Epsilonproteobacteria |
| 82 | 65564 | 66115 | + | Ornithine carbamoyltransferase (argF) | AAO57620 | 1,00E-40 | Gammaproteobacteria |
| 83 | 67582 | 66113 | - | Glycerol kinase (EC 2.7.1.30) | CAC49535 | 1,00E-124 | Alphaproteobacteria |

^a^ORF has highest homology to a protein from the SAR86-related environmental BAC clone EBAC31A08 [1]. Of the 67 ORFs annotated, 60%, 25%, and 15% were provisionally assigned based on the top BLAST hit to the *Gammaproteobacteria*, *Alphaproteobacteria*, and other prokaryotes, respectively.
