# Supplementary material for: New Insights into Metabolic Properties of Marine Bacteria Encoding Proteorhodopsins
Source: PLoS Biol. 2005 Jul 19;3(8):e273. doi: 10.1371/journal.pbio.0030273 (PMC1175822; doi:10.1371/journal.pbio.0030273)
Supplement: Supplementary file 6 [file pbio.0030273.st006.doc]

Table S6. List of genes on BAC clone MED35C06

| ORF | | | | Closest BLASTp hit | | | |
| --- | --- | --- | --- | --- | --- | --- | --- |
| Number | Start | Stop | Direction | Description | Protein_ID | E-value | Taxonomy of best blast hit |
| 1 | 473 |  | - | ABC transporter ATPase subunit | AAG10493 | 3,00E-52 | Gammaproteobacteria |
| 2 | 1483 | 473 | - | Esterase of alpha/beta hydrolase fold | AAG10492 | 2,00E-72 | Gammaproteobacteria |
| 3 | 1544 | 1981 | + | Thiol-disulfide interchange protein DipZ PA4845 | AAG08230 | 7,00E-06 | Gammaproteobacteria |
| 4 | 2006 | 2434 | + | AroD (aroD) | AAK03177 | 6,00E-41 | Gammaproteobacteria |
| 5^a^ | 2440 | 2877 | + | Biotin carboxyl carrier protein | AAQ62376 | 6,00E-36 | Gammaproteobacteria |
| 6 ^a^ | 2883 | 4229 | + | Acetyl-CoA carboxylase, biotin carboxylase | AAG10491 | 0.0 | Gammaproteobacteria |
| 7 ^a^ | 4222 | 6633 | + | Leucyl-tRNA synthetase (leuS) | AAG10490 | 0.0 | Gammaproteobacteria |
| 8 ^a^ | 6654 | 7088 | + | Hypothetical protein | - | 5,00E-06 | Gammaproteobacteria |
| 9 ^a^ | 7066 | 8028 | + | DNA polymerase III, delta subunit | AAG10489 | 2,00E-60 | Gammaproteobacteria |
| 10 ^a^ | 8863 | 8015 | - | D-alanine aminotransferase | AAG10488 | 2,00E-73 | Gammaproteobacteria |
| 11 ^a^ | 9998 | 8859 | - | Penicillin-binding protein 6 precursor | AAG10487 | 1,00E-146 | Gammaproteobacteria |
| 12 ^a^ | 10879 | 9998 | - | Membrane-bound lytic transglycosylase | AAG10486 | 1,00E-109 | Gammaproteobacteria |
| 13 | 11973 | 10879 | - | RodA (rodA) | AAK04009 | 8,00E-74 | Gammaproteobacteria |
| 14 ^a^ | 13804 | 11963 | - | Penicillin-binding protein 2 | AAG10485 | 0.0 | Gammaproteobacteria |
| 15 ^a^ | 14267 | 13980 | - | Hypothetical protein | AAQ62372 | 2,00E-12 | Gammaproteobacteria |
| 16 | 14612 | 14271 | - | Hypothetical protein XF2180 | AAF84979 | 7,00E-17 | Gammaproteobacteria |
| 17 ^a^ | 14693 | 15103 | + | Hypothetical protein | - | 1,00E-09 | Gammaproteobacteria |
| 18 ^a^ | 15692 | 15096 | - | Hypothetical protein | - | 6,00E-61 | Gammaproteobacteria |
| 19 ^a^ | 16986 | 15685 | - | UDP-N-acetylmuramate:L-alanyl-gamma-D-glutamyl-meso-diaminopimelate ligase | AAG10483 | 1,00E-123 | Gammaproteobacteria |
| 20 ^a^ | 17039 | 18289 | + | 6-phosphofructokinase | AAG10482 | 1,00E-171 | Gammaproteobacteria |
| 21 ^a^ | 18763 | 18290 | - | Hypothetical protein | - | 9,00E-34 | Gammaproteobacteria |
| 22 ^a^ | 21003 | 18775 | - | Topoisomerase IV subunit A | AAG10480 | 0.0 | Gammaproteobacteria |
| 23 ^a^ | 22896 | 21007 | - | Topoisomerase IV subunit B | AAG10479 | 0.0 | Gammaproteobacteria |
| 24 | 24343 | 22901 | - | Thiamin biosynthesis protein PA4973 (thiC) | AAG08358 | 0.0 | Gammaproteobacteria |
| 25 | 24654 | 24397 | - | Thiamin biosynthesis protein (thiC) | BAB05652 | 1,00E-07 | Firmicutes (Bacilli) |
| 26 ^a^ | 24854 | 26188 | + | Outer membrane protein TolC | AAG10478 | 1,00E-179 | Gammaproteobacteria |
| 27 ^a^ | 26196 | 27002 | + | Formamidopyrimidine-DNA glycosylase | AAG10477 | 3,00E-76 | Gammaproteobacteria |
| 28 ^a^ | 27491 | 27009 | - | Phosphopantetheine adenylyltransferase | AAG10476 | 8,00E-66 | Gammaproteobacteria |
| 29 | 28434 | 27532 | - | Transcription regulator | CAA61352 | 2,00E-48 | Gammaproteobacteria |
| 30 | 28535 | 30664 | + | Catalase (EC 1.11.1.6) | JS0520 | 0.0 | Firmicutes (Bacilli) |
| 31 | 30843 | 31595 | + | Proteorhodopsin | AAO73928 | 4,00E-89 |  |
| 32 | 32465 | 33529 | + | Haloalkane dehalogenase | AAK23159 | 2,00E-61 | Alphaproteobacteria |
| 33 ^a^ | 32483 | 31647 | - | Rossman fold oxidoreductase CsgA | AAG10473 | 3,00E-66 | Gammaproteobacteria |
| 34 | 33566 | 34177 | + | Glutathione S-transferase family protein | AAK25050 | 1,00E-23 | Alphaproteobacteria |
| 35 | 34204 | 35298 | + | Hypothetical protein bll1370 | BAC46635 | 9,00E-56 | Alphaproteobacteria |
| 36 | 35310 | 36341 | + | Glutathione S-transferase B1147A04.17 | BAB86200 | 0.052 | Eukarya |
| 37 ^a^ | 36357 | 37322 | + | 2-nitropropane dioxygenase | AAG10471 | 1,00E-165 | Gammaproteobacteria |
| 38 | 37436 | 38092 | + | Phosphatidylserine decarboxylase (EC 4.1.1.65) | AAL52672 | 6,00E-51 | Alphaproteobacteria |
| 39 | 38088 | 38768 | + | Phosphatidylserine synthase | AAK86871 | 6,00E-36 | Alphaproteobacteria |

^a^ORF has highest homology to a protein from the SAR86-related environmental BAC clone EBAC31A08 [1]. Of the 39 ORFs annotated, 77%, 13%, and 10% were provisionally assigned based on the top BLAST hit to the *Gammaproteobacteria*, *Alphaproteobacteria*, and other prokaryotes, respectively.
